# Deep Learning Algorithm for Automated Detection of Polycystic Ovary Syndrome Using Scleral Images

**DOI:** 10.3389/fendo.2021.789878

**Published:** 2022-01-27

**Authors:** Wenqi Lv, Ying Song, Rongxin Fu, Xue Lin, Ya Su, Xiangyu Jin, Han Yang, Xiaohui Shan, Wenli Du, Qin Huang, Hao Zhong, Kai Jiang, Zhi Zhang, Lina Wang, Guoliang Huang

**Affiliations:** ^1^Department of Biomedical Engineering, School of Medicine, Tsinghua University, Beijing, China; ^2^Reproductive Medicine Centre, Peking University Third Hospital, Beijing, China; ^3^National Engineering Research Center for Beijing Biochip Technology, Beijing, China

**Keywords:** polycystic ovary syndrome, deep learning, multi-instance learning, convolutional neural networks, sclera

## Abstract

The high prevalence of polycystic ovary syndrome (PCOS) among reproductive-aged women has attracted more and more attention. As a common disorder that is likely to threaten women’s health physically and mentally, the detection of PCOS is a growing public health concern worldwide. In this paper, we proposed an automated deep learning algorithm for the auxiliary detection of PCOS, which explores the potential of scleral changes in PCOS detection. The algorithm was applied to the dataset that contains the full-eye images of 721 Chinese women, among which 388 are PCOS patients. Inputs of the proposed algorithm are scleral images segmented from full-eye images using an improved U-Net, and then a Resnet model was applied to extract deep features from scleral images. Finally, a multi-instance model was developed to achieve classification. Various performance indices such as AUC, classification accuracy, precision, recall, precision, and F1-score were adopted to assess the performance of our algorithm. Results show that our method achieves an average AUC of 0.979 and a classification accuracy of 0.929, which indicates the great potential of deep learning in the detection of PCOS.

## Introduction

Polycystic ovary syndrome (PCOS) is known as one of the most common disorders among reproductive-aged women, affecting 6%–20% of premenopausal women worldwide (1). The cardinal symptoms of PCOS are ovarian dysfunction and androgen excess. Factors such as genetics, puberty, physiological changes, mental state, and environmental influences are widely considered to induce this syndrome. Patients with PCOS frequently demonstrate menstrual irregularities, hirsutism, obesity, insulin resistance, and cardiovascular diseases (2). Along with reproductive and metabolic disorders, a significant number of patients present psychological symptoms such as depression (3). Therefore, it is essential for the diagnosis and proper treatment of PCOS.

The diagnosis of PCOS is one of the most critical issues in the field of women’s healthcare. According to the clinical characteristics, PCOS is classified into different phenotypes. However, there is a controversy in the criterion of PCOS diagnosis. Among the criteria offered by various groups, the argument appears to affect the prevalence rates of PCOS. To ensure the accuracy of the initial diagnosis of PCOS, patients are supposed to have undergone ovarian ultrasonography. Some of them might even need to have venous sampling for metabolic evaluation. It is estimated that the average cost of initial diagnosis and evaluation of PCOS is $740 (4), which is a significant financial burden.

With the rapid development of artificial intelligence, using machine learning and deep learning to assist with PCOS detection has attracted much more attention. For instance, the authors of (5) applied a machine learning algorithm to the clinical parameters of PCOS to select the most contributed features and predicted PCOS patients. They achieved an accuracy of 91.01% using random forest and logistic regression. The authors of (6) collected gene biomarker data from PCOS patients and normal groups and then they proposed a model based on random forest and artificial neural network to classify PCOS samples and normal samples. They achieved an AUC of 0.7273 in one of their datasets.

The sclera, a visible part of the ocular surface, is a dynamic tissue that is constantly remodeled (7) and may reflect human health. A yellowish staining of the sclera may be associated with liver disease (8). It has been discovered that the abnormal vasodilation of the conjunctival blood vessels leads to the appearance of redness sclera (9). Researchers have found that some female sex steroids have had ocular effects in recent years, and PCOS leads to physiological changes in the eyes (10). Bonini et al. (11) reported that of 62 young women diagnosed with PCOS, 94% were accompanied by ocular disease. Compared with other women, PCOS patients had more severe conjunctival congestion, dry eyes, and itching eyes. However, little work has been reported in using deep learning on scleral images to assist with the screening of PCOS groups.

In this paper, we explored a novel method for the screening of PCOS using scleral images. The proposed method is composed of image preprocessing, features extraction, and classification steps based on deep learning. We used an improved U-Net embedded with an attention module to segment the sclera from full-eye images, a Resnet18 to extract deep features, and a multi-instance learning model to classify PCOS and normal samples. Results show that our non-invasive screening method achieved a mean AUC of 98%, a mean accuracy of a dataset that contains 721 subjects.

## Materials and Methods

### Data Acquisition and Dataset Establishment

**Figure 1:**
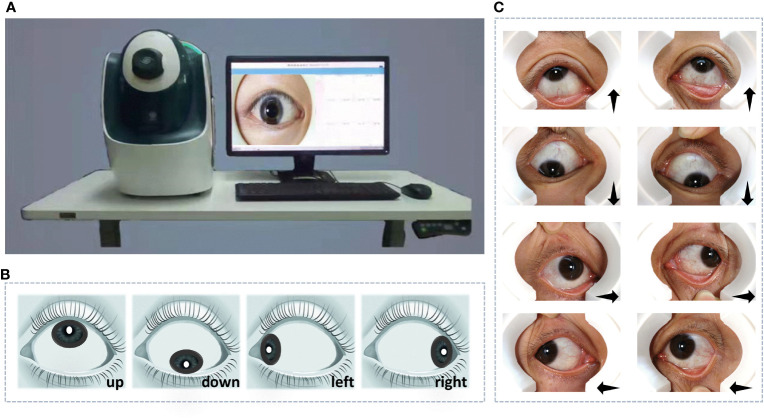
The device for data acquisition and data collected for experiments. **(A)** The specially designed device. **(B)** The diagram of reflection-shadows-free scleral images. **(C)** Images of the left and right eyes with eyeballs rotating in different directions: up, down, left, and right.

Our method was developed using data collected by a specially developed device described in (12) (**Figure 1A**), which was designed for obtaining reflection-shadows-free scleral images. To obtain information on the sclera completely, we captured images of the eyeball rotating in different directions: up, down, left, and right. Eight scleral images, including the right and left eyes, were collected for every patient (**Figure 1B**). This process is fast, low-cost, and painless.

We recruited more than 800 women of childbearing age as subjects. Through data cleaning, the blurred and incomplete sclera images were removed and there 721 subjects remained, including 388 PCOS patients diagnosed by medical professionals. The data were mainly collected in Peking University Third Hospital (Beijing, China) from 2017 to 2019 and in part collected in community checkup using the same instrument at the same time. In order to avoid the influence of different data collection times and different operators, we have performed histogram equalization for all images to avoid systematic data deviation. Subjects were required to rotate their eyeballs up, down, left, and right, respectively (**Figure 1C**), and the captured eight scleral images were considered a data bag for the corresponding subject. Data bags were labeled as positive for subjects with PCOS, while others were labeled as negative. The division only counted on the presence of PCOS and did not exclude other diseases.

To remove the potential dependency of classification performance on dataset partition, we performed a 5-fold cross-validation procedure to split data: 145 of the 721 data bags in the dataset were separated for testing, while the rest were used as training set to develop the algorithm. Namely, two subsets were split according to subjects instead of scleral images, and images from the same subjects can only be present either in the training set or in the test set. Named ScleraSet, this dataset was established for the detection of PCOS.

Moreover, we recruited a wider range of subjects, including men and women of non-reproductive age, and collected scleral images as the training set for the sclera segmentation network. To reasonably evaluate the performance of the sclera segmentation network in ScleraSet, images were randomly selected from ScleraSet as test set. The training set contains 1,736 images, and the test set includes 340 images.

### Image Preprocessing

Aimed at enhancing the performance of classification algorithms, a standardized image-preprocessing procedure was established. This procedure consisted of two main steps, which were vital for developing a PCOS detection algorithm.

### Image Enhancement

Depending on the healthy state of subjects, characteristics such as blood vessel color and macula may play an important role in this scleral image-based classification problem. To improve the performance of classification algorithms, it is necessary to enhance features on scleral images and also to minimize the influence of imaging light conditions.

Firstly, blur images with a Gaussian blur function. The principle is to carry out the convolution operation of the Gaussian function to the image and carry out the Gaussian transformation to every pixel in the image. After blurring, the high-frequency components of the image, such as vessels and spots, remain visible in the image. Then, blend the blurred image with the original one and give higher weights to the high-frequency components to achieve image enhancement.

### Sclera Segmentation

As shown in **Figure 1C**, the original images captured by the specially developed device contained full-eye information along with some skin around eyes. Hence, it is essential to segment the sclera from full-eye images to avoid the interference of invalid areas. With the rapid development of artificial intelligence, segmentation methods based on deep learning becomes increasingly popular. Compared with traditional segmentation algorithms, Convolutional Neural Networks (CNNs) can achieve pixel-level segmentation by extracting features from the input original image without manual feature selection. For accurate sclera segmentation, we used an improved U-Net model (13), which is an embedded attention module contributed by Woo et al. (14). A previous study suggested (15) that such architecture has great advantages in sclera segmentation. To ensure the simplicity of network training and accelerate the process, batch normalization (16) was implemented after every convolutional layer.

**Figure 2:**
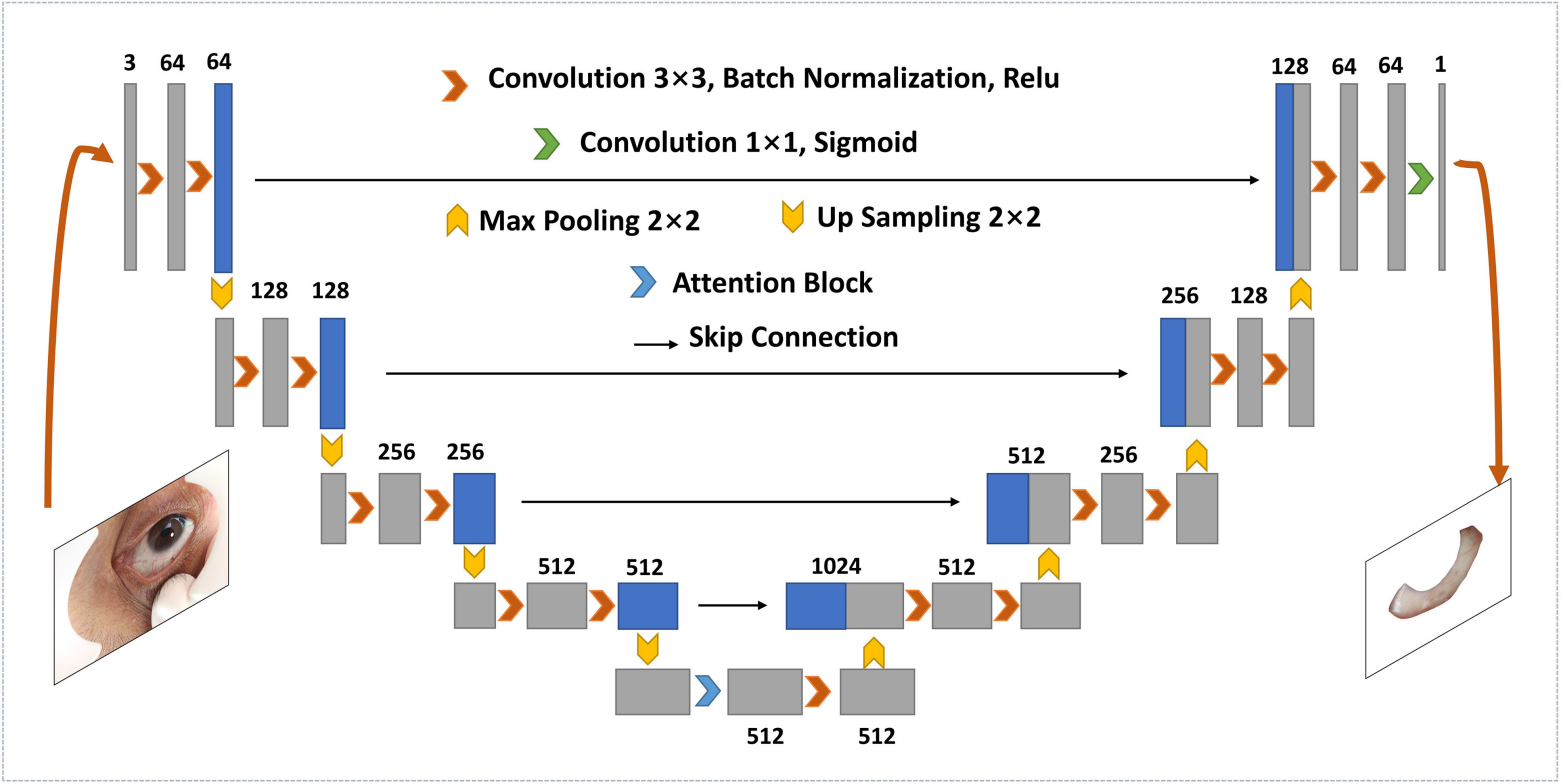
Overview of the scleral segmentation model embedded with attention module.

The architecture of the segmentation network is shown in **Figure 2**, which consists of a classic U-Net network and a convolutional block attention module (CBAM). It is a U-shaped structure, containing a contracting path and an expansive path. The former is equivalent to an encoder and composed of eight 3×3 convolutional layers and four 2 × 2 max-pooling layers with stride 2. For each convolutional layer, one batch normalization layer and one rectified linear unit (ReLU) follow. As the depth of the network increases, the number of channels in different convolutional blocks increases, while the output size of feature maps decreases. At the bottom of the U-shaped structure, where the contraction and expansion paths intersect, CBAM is embedded. The attention module is divided into a spatial attention module and a channel attention module. The former is used to encode the importantly segmented spatial information, while the latter is used for the category information. At the expansion path, convolutional units consist of three up-sampled convolutional layers and one convergence layer, where the convergence layer connects the feature map from the contraction path and the output from the upper convolutional unit. In the end, the output convolutional layer carries a sigmoid activation function that generates prediction distribution maps.

### Deep Learning Architecture

**Figure 3:**
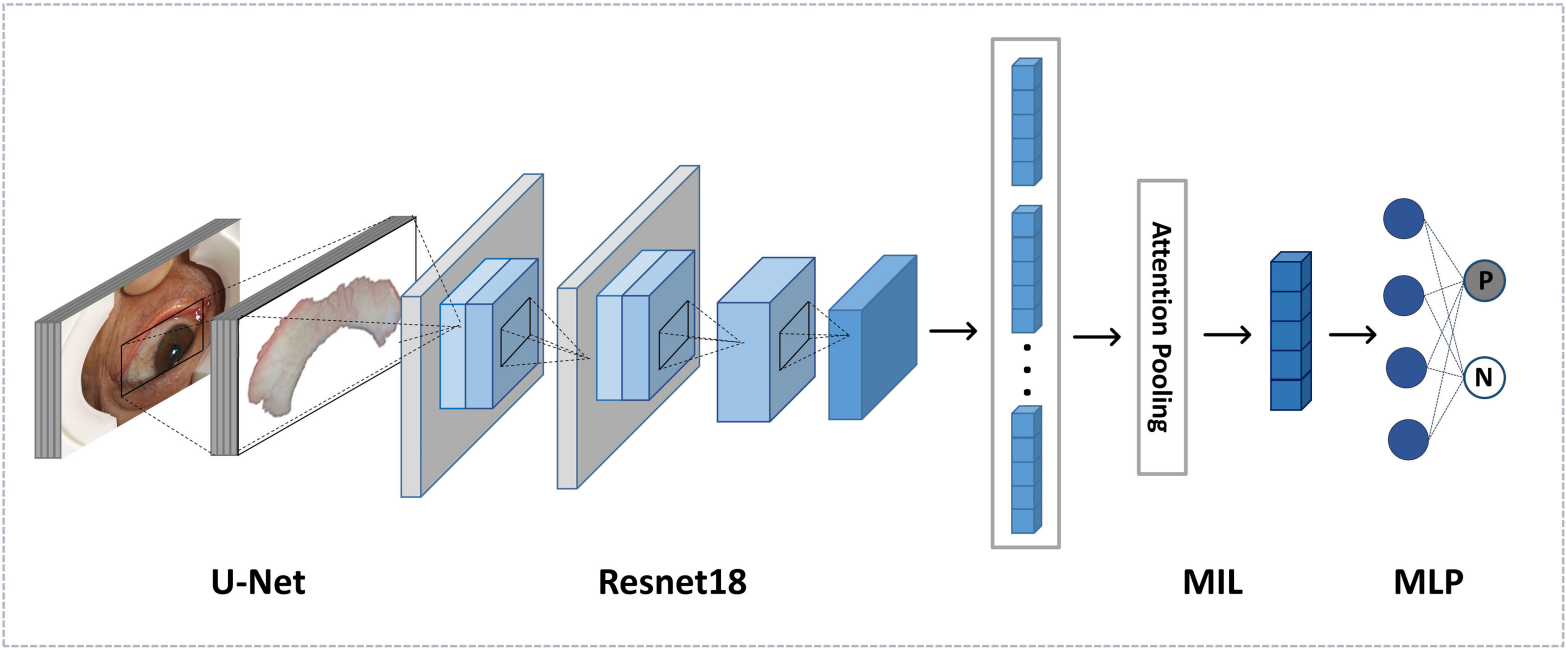
Overview of the framework of the diagnosis algorithm.

Based on CNN, our proposed PCOS deep learning architecture is composed of feature extraction and classification. The former was used to extract important deep features from sclera images, while the latter is a multi-instance learning (MIL) model. Inputs are processed images, and outputs of the last softmax layer reveal the prediction of PCOS. The overview of the proposed deep learning architecture is shown in **Figure 3**.

### Feature Extraction

Feature extraction based on deep learning can achieve relatively great classification results by comparison with traditional handcrafted features (17, 18). We applied Resnet to our feature extraction network, which was proposed by He et al. in 2016 (19) and outperformed previous CNNs in a great number of applications about images. The backbone of feature extraction is the Resnet18 model.

The feature extraction network is composed of five residual learning groups and eight residual connections, similar to Resnet18, except for the fully connected layers. There is one 7 × 7 convolution layer in the first group, while the remaining groups contain four convolution layers each. After each convolutional layer, ReLU is adopted as the activation function. The input of the network is an RGB scleral image of 512 × 512. After the average pooling layer, the output feature size is 512.

### Classification

For every subject, eight scleral images were captured to avoid any information missing. Instead of making decisions on single image, we considered all images of every subject, which is more reasonable and appropriate. To address the multiple instances issue, we used a multi-instance (MIL) model.

For each subject, all scleral images ${\text{x}}_{\text{i}}={\left\{{\text{s}}_{\text{k}}\right\}}_{k}^{8}$ were fed into our feature extraction network to obtain feature vectors ${\left\{{\text{p}}_{\text{k}}\right\}}_{k}^{8}$ . There are two steps for the MIL model to give a prediction. The first is to aggregate all feature vectors ${\left\{{\text{p}}_{\text{k}}\right\}}_{k}^{8}$ from the same subject by an aggregation function *g*. Later, outputs ${\left\{g({\text{p}}_{\text{k}})\right\}}_{k}^{8}$ were fed into a multi-layer perceptron (MLP) to obtain the bag probability.

The commonly used aggregation function is max-pooling or average pooling, but we used an attention-based MIL pooling proposed by Ilse et al. (20) to optimize our model. This MIL pooling is:


(1) $$m=\sum_{k=1}^{8}\frac{\exp\{W^{T}\tanh(Vp_{k}T)\}}{\Sigma_{o=1}^{8}\exp\{W^{T}\tanh({\text{Vp}}_{o}T)\}}pk.$$

W and V are parameters of network.

Inputs of the MIL model are feature vectors of size 512, and outputs are the bag label for each subject. “Positive” represents normal samples, while “negative” represents PCOS samples.

## Results

We performed the proposed detection algorithm on ScleraSet mentioned previously. Furthermore, to explore the feature extraction performance and the positive effects of attention mechanism applied in feature extraction network, we compared the detection performance of different CNNs including Inception V3, Vgg16, and Vgg19 as feature extraction networks.

To assess the performance of our proposed algorithm, we utilize AUC, accuracy, precision, recall, and F1-score as the evaluation indices. Firstly, the receiver operating characteristic (ROC) curve was plotted to present the detection performance. ROC curve is a graph that plots the true-positive rate and false-negative rate. Later, calculate the area under the ROC curve (AUC). According to the ROC curve, we searched the optimal threshold on the basis of Youden’s *J* statistic. Accuracy, precision, recall, and F1-score were all computed at the optimal threshold. Expressions of these indices are shown as follows, where TP denotes True Positive, TN denotes True Negative, FN denotes False Negative, and FP denotes False Positive:


(2) $$Accuracy=\frac{TP+TN}{TP+TN+FN+TN}$$

(3) $$Precision=\frac{TP}{TP+FP}$$

(4) $$Recall=\frac{TP}{TP+FN}$$

(5) $$F1-score=2\frac{Precision\times recall}{Precision+recall}$$

Adaptive Moment Estimation (Adam) was used as optimizer, and the base learning rate is 0.0001. For different experiments, the optimal learning rate was chosen for minimal errors and we obtain results using the best learning rate. The loss function we adopted was cross entropy; training epochs depended on the convergence time. All experiments were conducted on PyTorch with two Tesla V100 Graphics Processing units (GPUs).

**Figure 4:**
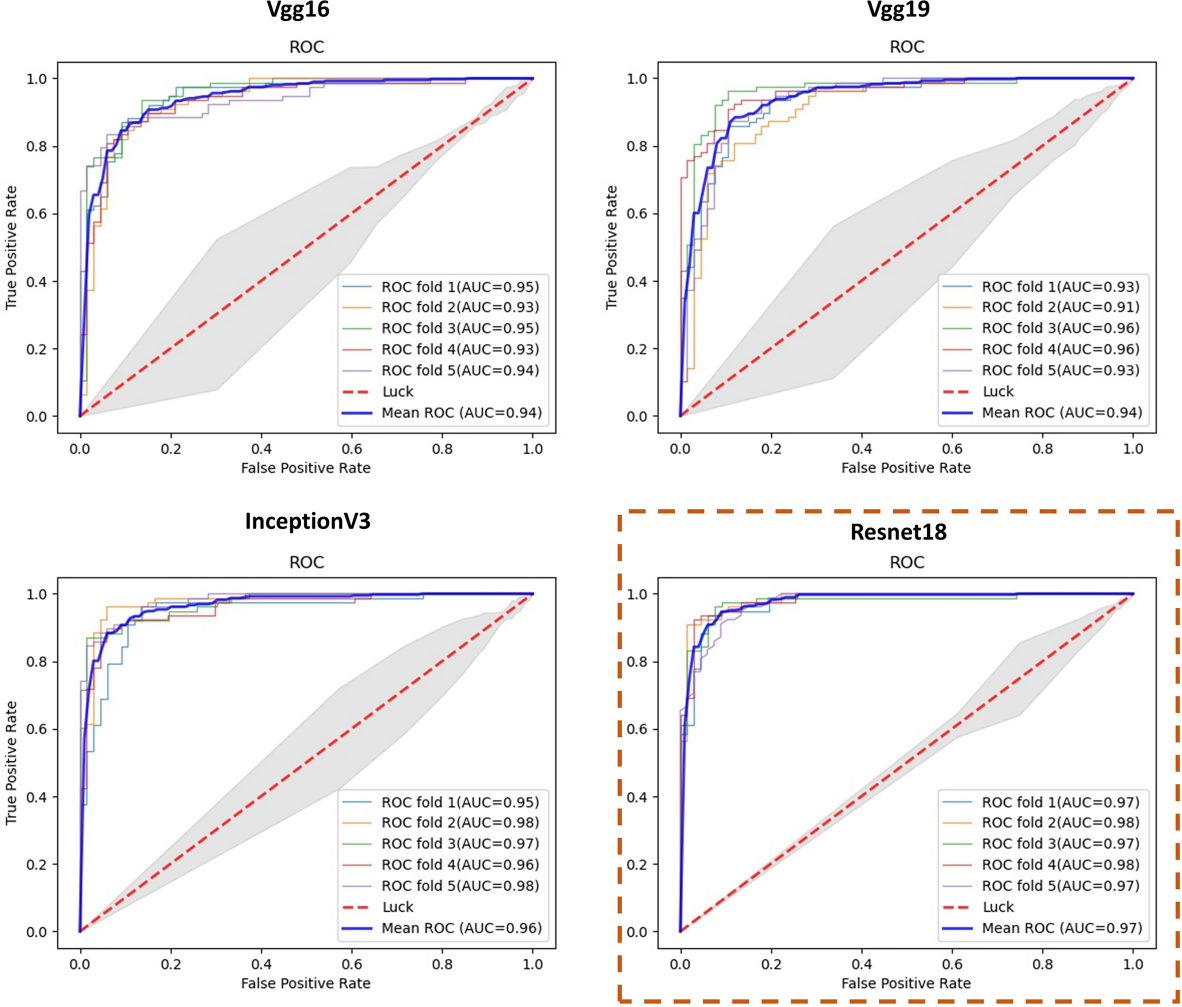
ROC curves of classification results using different feature extraction networks.

Results of the test sets in 5-fold cross-validation experiments are shown in **Table 1** and **Figure 4**. As shown in **Table 1**, our proposed deep learning architecture achieved a mean AUC of 0.979, a mean accuracy of 0.929. In contrast, when VGG16, Vgg19 (21), and inceptionV3 (22) were chosen as the feature extraction network, the mean AUC was 0.942, 0.940, and 0.967, respectively. **Figure 4** shows the ROC curve of all experiments. The closer the ROC curve gets to the top left corner of the ROC box, the better the performance the model achieves. Compared with other curves, the curve of the model with Resnet is closer to the top-left point, which suggests that the performance of the model with Resnet has excellent sensitivity and specificity.

**Table 1:** Results of comparison between different feature extraction networks in 5-fold cross-validation experiments.

Task	AUC	Accuracy	Precision	Recall	F1-score
VGG16	0.942 ± 0.007	0.871 ± 0.005	0.885 ± 0.005	0.874 ± 0.005	0.879 ± 0.005
VGG19	0.940 ± 0.019	0.877 ± 0.031	0.892 ± 0.027	0.876 ± 0.031	0.884 ± 0.029
Inception	0.967 ± 0.012	0.913 ± 0.014	0.924 ± 0.013	0.912 ± 0.013	0.918 ± 0.013
**Resnet**	**0.979 ± 0.003**	**0.929 ± 0.007**	**0.940 ± 0.006**	**0.928 ± 0.006**	**0.934 ± 0.006**

The bold values means that Resnet had the best performance.

## Discussion

Scleral images are usually used in the field of biometric recognition (23–25). However, there are rare studies about the disease detection potential of sclera images. In this manuscript, we proposed a novel algorithm to help with the detection of PCOS. To the best of our knowledge, this is the first attempt to explore the relationship between scleral images and PCOS utilizing deep learning.

This manuscript proposes a non-invasive method for the automatic detection of polycystic ovary syndrome based on scleral images. The method consisted of image processing and deep learning techniques to classify scleral images of general subjects and PCOS patients. After simple image preprocessing, we used an improved U-Net model for the rapid sclera segmentation. This model achieved an intersection over union (IoU) of 0.853, which was revealed to be adequate for this project. As for PCOS detection, a feature extraction network was used for obtaining important deep features in the sclera, and later a MIL model was applied to the final classification. The PCOS detection algorithm proposed in this paper achieved good AUC, accuracy, and other indicators. According to the experiment results, Resnet18 obtains a top performance in the comparisons of VGG16, VGG19, and inception V3. There is an apparent enhanced performance using Resnet as the feature extraction network.

**Figure 5:**
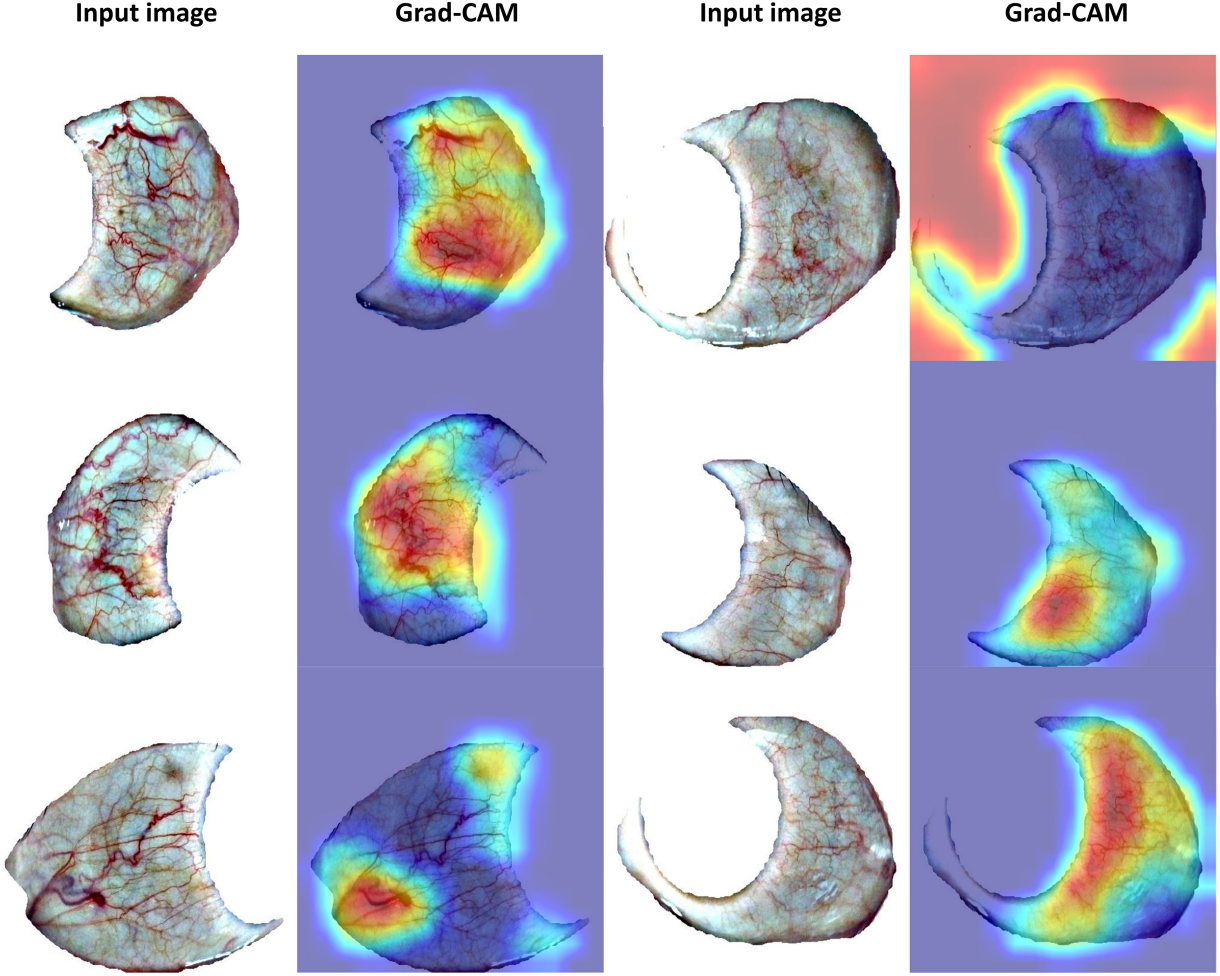
Visualization results with Grad-CAM.

Furthermore, this manuscript focuses more on algorithmic research than pathology. To visualize what our model has learned, we used Grad-CAM (26), a popular CNN explanation tool to highlight the features that is important for PCOS detection. Grad-CAM utilizes the last convolutional layers and marks the decisive features by a heatmap. Some positive samples with Grad-CAM visualization are shown in **Figure 5**. We found that our model focused on thick blood vessels, foggy blood vessels distributed over a large area, and some kinds of spots (the second column in **Figure 5**), which seems to verify the findings of other studies (10, 11) that PCOS probably causes the changes of blood vessels in the sclera because of sex steroids disorder. However, some highlighted regions can be confusing (the fourth column in **Figure 5**), since our work is exploratory and more physiological study is needed.

Although the deep learning algorithm performed well on the dataset we collected, there are limitations in our work and problems to be solved in the future. Firstly, our dataset is not large enough and lacks other data for evaluating the network’s generalization ability. Heterogeneity is one of the critical features of PCOS; hence, a larger dataset is supposed to be collected from a wider range of groups. What is more, on account of data masking, all the information we obtained is whether subjects were PCOS patients. Therefore, our experiments did not take into account the effects of factors such as specific medical condition, race, and geography. Besides, some visualization results seem difficult to explain. Future work can establish pathological models and consider studying the intrinsic connections between changes in the sclera and the physiological changes triggered by PCOS. Then, combine these connections with the algorithm to enhance the interpretability of the algorithm and give doctors more explicit information to assist in detection.

## Conclusion

The screening of PCOS has received considerable critical attention. For this issue, the present study was designed to explore a non-invasive method to help with PCOS detection. Our work shows that the proposed algorithm obtains a significant classification performance (mean AUC of 0.978), which indicates that deep learning might be a powerful tool for PCOS detection. Besides, experiment results may imply the outstanding potential of applying scleral images to disease detection. The combination of artificial intelligence and features extracted from scleral images may become a valuable research field.

## Data Availability Statement

The raw data supporting the conclusions of this article will be made available by the authors, without undue reservation.

## Ethics Statement

The studies involving human participants were reviewed and approved by the Ethics Committees of the Peking University Third Hospital and Tsinghua University. Written informed consent for participation was not required for this study in accordance with the national legislation and the institutional requirements.

## Author Contributions

WL undertook the main work of coding, analyzing data, and writing the manuscript. YS had key contribution in data collection. RF provided some suggestions on the algorithm. YS, XJ, QH, and HZ assisted in segmenting the scleral image. XL, XS, WD, and KJ assisted in collecting data. ZZ, LW and GH are the corresponding authors. All authors contributed to the article and approved the submitted version.

## Funding

The authors thank all funding support from the National Key Research and Development Program of China (2018YFA0704000), the National Natural Science Foundation of China (61927819, 81827808, and 62105177), the Tsinghua University Spring Breeze Fund (2020Z99CFG011), the Beijing Lab Foundation, and the Tsinghua Autonomous Research Foundation (20194180031 and 20201080058).

## Conflict of Interest

The authors declare that the research was conducted in the absence of any commercial or financial relationships that could be construed as a potential conflict of interest.

## Publisher’s Note

All claims expressed in this article are solely those of the authors and do not necessarily represent those of their affiliated organizations, or those of the publisher, the editors and the reviewers. Any product that may be evaluated in this article, or claim that may be made by its manufacturer, is not guaranteed or endorsed by the publisher.
